# Bioluminescence-mediated longitudinal monitoring of adipose-derived stem cells in a large mammal *ex vivo* organ culture

**DOI:** 10.1038/srep13960

**Published:** 2015-09-09

**Authors:** Mirte Peeters, Sjoerd van Rijn, Pieter-Paul A. Vergroesen, Cornelis P. L. Paul, David P. Noske, W. Peter Vandertop, Thomas Wurdinger, Marco N. Helder

**Affiliations:** 1 Department of Orthopedic Surgery, VU University Medical Center, MOVE Research Institute Amsterdam, Amsterdam, The Netherlands; 2 Department of Neurosurgery, VU University Medical Center, Amsterdam, The Netherlands; 3 Neuroscience Center, Department of Neurology, Massachusetts General Hospital and Neuroscience Program, Harvard Medical School, Boston, MA, USA

## Abstract

Recently, *ex vivo* three-dimensional organ culture systems have emerged to study the physiology and pathophysiology of human organs. These systems also have potential as a translational tool in tissue engineering; however, this potential is limited by our ability to longitudinally monitor the fate and action of cells used in regenerative therapies. Therefore, we investigated luciferase-mediated bioluminescence imaging (BLI) as a non-invasive technique to continuously monitor cellular behavior in *ex vivo* whole organ culture. Goat adipose-derived stem cells (gADSCs) were transduced with either Firefly luciferase (Fluc) or *Gaussia* luciferase (Gluc) reporter genes and injected in isolated goat intervertebral discs (IVD). Luciferase activity was monitored by BLI for at least seven days of culture. Additionally, possible confounders specific to avascular organ culture were investigated. Gluc imaging proved to be more suitable compared to Fluc in monitoring gADSCs in goat IVDs. We conclude that BLI is a promising tool to monitor spatial and temporal cellular behavior in *ex vivo* organ culture. Hence, *ex vivo* organ culture systems allow pre-screening and pre-validation of novel therapeutic concepts prior to *in vivo* large animal experimentation. Thereby, organ culture systems can reduce animal use, and improve the speed of innovation by overcoming technological, ethical and financial challenges.

Recently, *ex vivo* three-dimensional (3D) whole organ culture systems have emerged as means with which to study the physiology and pathophysiology of human organs and tissues. Pluripotent human stem cells and their self-organizing capacities have been used to develop bowel, kidney, brain, retina and liver-bud organoids^1^,^2^. Additionally, animal and human tissue explants are used in long-term *ex vivo* culture. Intestines, intervertebral discs, pancreas and skin explants have been cultured to study biological and pathological processes^2^,^3^,^4^,^5^,^6^. Subsequently, these 3D culture systems are increasingly used to study therapies, for instance in prostate cancer^7^.

In addition to traditional drug-based approaches, tissue engineering aims to regenerate damaged or degenerated tissue and organs using cells or regeneration inducting factors. However, the fate and action of these cells after implantation are often hard to determine, and research will therefore benefit from longitudinal monitoring of cellular behavior. Luciferase-mediated bioluminescence imaging (BLI) is a non-invasive imaging technique which allows real-time *in vivo* monitoring of location and proliferation of luciferase-expressing cells^8^,^9^. BLI is based on the emission of photons produced during substrate conversion by luciferase enzymes. These photons can be detected by a cooled charged coupled device (CCD) camera through several millimeters of tissue. The considerable penetration depth, including cartilage and bone, and the higher signal-to-noise ratio of the BLI technique are marked advantages compared to conventional fluorescent cell viability assays which require laser excitation for cell visualization. Different dye methods used to determine cell viability require lysis or fixation of cells or tissue. This results in termination of the experiment, and therefore impedes longitudinal monitoring of cell viability within a single sample. Finally, BLI has been shown to be highly correlated to the above mentioned cell viability assays (for both Fluc and Gluc) and is therefore a well-established method for cell viability monitoring^10^,^11^. These advantages highlight the potential of BLI for longitudinal evaluation of cellular therapies in *ex vivo* tissue culture^12^.

*In vivo*, bioluminescent imaging is only performed in small animal models^13^,^14^,^15^,^16^,^17^. Results found in small animals are often difficult to extrapolate to the human situation since they do not properly reflect the human physiology and/or dimensional conditions^18^. On the other hand, the size of large mammals impedes *in vivo* bioluminescent imaging due to the current penetration depth of BLI. The use of BLI in *ex vivo* organ culture systems offers a potential solution to overcome this problem.

Recently, we developed an *ex vivo* loaded disc organ culture system (LDCS), to study the physiology of intervertebral discs (IVD) and their response to mechanical loads^4^,^19^. This system allows us to investigate the process of intervertebral disc degeneration, identified as an important etiological factor of low back pain^20^,^21^. In the LDCS goat IVDs are used as they are a representative model for the human IVD^22^. In addition, the LDCS allows for preclinical *ex vivo* evaluation of therapies aimed at tissue regeneration prior to actual *in vivo* animal experimentation^23^, without the problems related with the translation of small animal research to humans.

Mesenchymal stem cells (MSCs) are excellent candidates for regenerative therapies because of their relatively young age and multi-differentiation potential. Furthermore, studies directly comparing young and adult chondrocytes with age-matched MSCs showed that - based on aggrecan ultrastructure, ECM composition, and cellular proliferation - MSCs produce a superior cartilage-like neo-tissue compared to either young or adult chondrocytes^24^,^25^. Previous research showed successful luciferase reporter transductions of MSCs obtained from humans and animals^13^,^26^,^27^,^28^. *In vivo*, MSCs show promising results when transplanted into rat and rabbit IVDs, and these cells survive, differentiate, and increase the production of functional extracellular matrix, thereby arresting the degeneration process^29^,^30^,^31^. Furthermore, MSCs are easy to harvest in large quantities from adipose tissue and are therefore often used for tissue engineering purposes^32^.

The aim of the current study has been to evaluate the use of luciferase-mediated bioluminescence imaging for longitudinal monitoring of adipose derived mesenchymal stem cells injected in large mammal IVDs, cultured in an *ex vivo* organ culture system. We investigated the feasibility of commonly used luciferases: the intracellular Firefly luciferase (Fluc) and the naturally secreted *Gaussia* luciferase (Gluc) which can be detected in the cell as well as in the extracellular environment^33^. Additionally, possible confounders specific to avascular organ culture were investigated.

## Material and Methods

### Cell Culture

Mesenchymal stem cells were isolated from subcutaneous adipose tissue, collected from skeletally mature Dutch female milk goats (age 3–5 years). Isolation of the stem cells was performed as described by Zuk *et al.*^34^. The research protocol was approved by the scientific board and the animal ethics committee of the VU University, and is in accordance with national guidelines and regulations. In brief, between 50 and 100 grams of tissue was harvested subcutaneously from the para-vertebral area. Adipose tissue was minced, washed in phosphate buffered saline (PBS, Life Technologies), and digested using 1 U Liberase TM (Roche) per gram of tissue in PBS for 60 minutes at 37 °C under gentle shaking conditions. The digested tissue was filtered through a 100 μm mesh filter to remove residual extracellular matrix and to obtain a single cell suspension. The resulting single cell suspension, containing adipose stem cells, was diluted in Dulbecco’s modified Eagle’s medium (DMEM, Life technologies) containing 10% Hyclone fetal bovine serum (FBS, Thermo Scientific), Penicillin (10,000 units/ml), streptomycin (10 mg/ml) and amphotericin B (25 μg/ml) (1% PSF; Sigma Aldrich) and plated at 1–4 × 10^6^ nucleated cells/cm^2^. Twenty-four hours after seeding, culture medium was refreshed and goat adipose derived stem cells (gADSCs) were obtained by expanding the plastic adherent cells. As a positive control, U87 glioblastoma cells were cultured in DMEM complemented with 10% FBS and 1% PSF. All cells were grown in a humidified incubator at 37 °C and 5% CO_2_. Medium was refreshed twice a week and upon reaching near-confluence cells were detached using 0.25% trypsin/0.1% EDTA (Life Technologies) and diluted for further culture. For all experiments involving gADSCs, we used early passage gADSCs (≤passage 4).

### Isolation of intervertebral discs

Spines of skeletally mature 3-to-5-year-old Dutch female milk goats were obtained from a local abattoir. Lumbar IVDs were dissected within 24 hours after sacrifice, as described by Paul *et al.*^4^. Briefly, we dissected the intervertebral discs (T13-L1 to L5-L6) including the adjacent cartilaginous endplates under sterile conditions using an oscillating surgical saw. Excess ligaments and posterior elements were removed, and IVDs were cleaned to remove blood, and washed in PBS supplemented with 1% PSF. Using these IVDs, we bypassed the technological, ethical and financial challenges that are involved with laboratory animal experimentation.

### Lentivirus production and transduction

**Figure 1:**
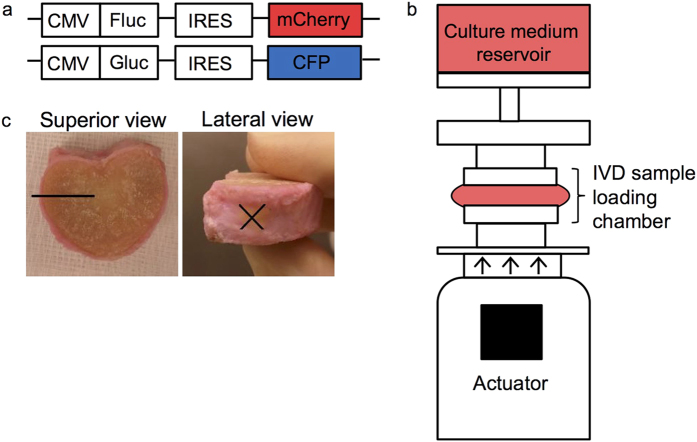
Methods for goat adipose derived stem cell imaging *ex vivo*. (**A**) Schematic depiction of the Firefly luciferase (Fluc) reporter co-expressing mCherry and of the *Gaussia* luciferase (Gluc) reporter co-expressing Cerulean fluorescent protein (CFP). (**B**) Schematic overview of the loaded disc culture system (LDCS) that we use to simulate the normal physiological conditions of the intervertebral disc (IVD). For details see Paul *et al.* 2012^4^. **(C)** Superior and lateral view of the goat IVD. Location of cell injection is depicted by “X” and needle track is shown by the black line.

The Gluc and Fluc lentivirus vector reporters co-encode the fluorescent genes Cyan Fluorescent Protein (CFP) and mCherry, respectively, as described elsewhere (Fig. 1a)^10^,^35^. To produce lentivirus particles, the lentivirus reporters were co-transfected with a third generation lentiviral packaging plasmid mix (pMDLg/pRRE, pRSV-Rev and pMD2.G, Addgene) in HEK293T cells using Lipofectamine 2000 (Life Technologies), according to manufacturer’s guidelines. Lentivirus particles were harvested two and three days after transfection and cell debris was spun down for five minutes at 1000 × G. We plated low passage gADSCs and U87 cells in 25 cm^2^ culture flasks and when the cultures reached ~60% confluence we transduced them with the Gluc-CFP or the Fluc-mCherry bioluminescent reporter gene with a multiplicity of infection 10–20. Transfection was achieved by washing the cells with PBS and subsequently adding 3 ml of lentivirus-conditioned medium. After an incubation period of four hours for the gADSCs, or overnight for the U87 cells we removed the lentivirus conditioned medium and washed the cells with PBS. Finally, we added fresh culture medium. We verified transduction efficiency by fluorescence microscopy (Leica) of the co-expressed fluorescent reporters. gADSCs expressing Gluc and CFP are further mentioned as gADSC-GC. gADSCs expressing Fluc and mCherry are referred to as gADSC-FM. For the U87 cells the same terminology is used: U87-GC and U87-FM cells.

### *In vitro* bioluminescent activity measurements of Gluc and Fluc

To measure Fluc activity, we lysed the gADSC-FM with 100 μl Reporter Lysis Buffer (Promega) and three freeze-thaw cycles. Aliquots of 10 μl lysate were mixed with 40 μl of Luciferase Assay Reagent (Promega). Luciferase activity was measured immediately after mixing for two seconds using a tube luminometer (Berthold Technologies) and expressed as Relative Light Units (RLU). For Gluc activity, the Gluc substrate coelenterazine was prepared freshly by diluting the coelenterazine stock (Nanolight, 5 mg/ml in methanol) 1:1000 in PBS + 0.1% Triton X-100, to a final concentration of 5 μg/ml. A total of 10 μl of Gluc conditioned medium was collected from the cell culture of gADSC-GC cells, 40 μl coelentrazine was added and activity was measured as described above. To determine luciferase activity over time, 100, 1000 or 10,000 gADSCs were plated in a 24 wells plate (n = 3 per condition) and luciferase activity was measured 1, 4 and 7 days after initial plating.

### *Ex vivo* bioluminescent imaging

A total of 0.5 × 10^6^ gADSC-FM, gADSC-GC, U87-FM or U87-GC cells was suspended in 50 μl DMEM. Cells were injected in the nucleus pulposus (NP) via the left lateral side, through the annulus fibrosus of the IVD using a 29G needle of 1 cm length (Fig. 1c). Pilot experiments to image Gluc and Fluc expressing cells in the IVD were performed to determine suitable substrate concentration and imaging conditions. We imaged Fluc activity by injecting 50 μl of 30 mg/ml d-luciferin (Goldbio Technology) dissolved in PBS into the NP prior to each BLI measurement. Gluc activity was measured by injecting 50 μl of 1 mg/ml coelenterazine substrate in PBS + 0.1% Triton X100 prior to each BLI measurement. For both groups, luciferase activity was measured directly after substrate administration using the IVIS CCD camera (Perkin Elmer). Exposure times were 10 minutes for IVDs injected with Fluc reporter cells, and 30 seconds for IVDs injected with Gluc reporter cells. As a negative control we imaged IVDs injected with Fluc or Gluc substrate and as a positive control the high Fluc/Gluc expressing U87 glioma tumor cells are run alongside in parallel experiments for *ex vivo* imaging of cells in IVDs. We analyzed the images with Living Image software (Perkin Elmer) and activity was defined as the sum of all photons/second (p/s) detected within a defined region of interest (ROI).

### Repetitive substrate addition to reporter expressing cells

We conducted the following experiments to determine the possible toxic effect of repetitive addition of the luciferase substrates on gADSC (*i.e.* d-luciferin for gADSC-FM and coelenterazine for gADSC-GC). gADSC-FM were seeded at 5000 cells/cm^2^ in complete culture medium, and bioluminescence activity was assessed after 1 and 4 days. Fluc substrate, d-luciferin, was diluted in complete pre-warmed (37 °C) culture medium (to a concentration of 150, 250, 500 and 1000 μg/ml d-luciferin). The concentrations were selected based on the by Xenogen^36^ advised concentration for *in vitro* use of d-luciferin (150 μg/ml) and the amount of d-luciferin we injected in the IVD during the *ex vivo* experiments (1000 μg/ml). Directly before imaging, culture medium was aspirated from the cells, and the d-luciferin-containing medium was added to the cells. Fluc activity was measured by determining photon count using a CCD camera for 30 seconds. Signal intensities were defined as the sum of all photons/second detected within a single well and values were normalized to the total amount of DNA per well. DNA content was quantified using the CyQuant GR (Life Technologies) assay according to manufacturer’s protocol. Cells were washed with PBS and lysed with 200 μl sterile Milli-Q and a freeze-thaw cycle. For the cells receiving the substrate repetitively, medium containing d-luciferin was not refreshed after imaging at day 1. This way, we mimicked the *ex vivo* situation where d-luciferin is contained in the avascular IVD and is only partially eliminated through diffusion, which is a similar process to the slow nutrient exchange.

To determine the effect of repeated coelenterazine substrate administration on gADSCs, we cultured gADSC-GC at 5000 cells/cm^2^ and measured Gluc activity for 4 consecutive days. We added either coelenterazine (1 mg/ml or 5 μg/ml, *i.e. ex vivo* and *in vitro* concentrations), or methanol (equivalent amount to coelenterazine 1 mg/ml), dissolved in complete culture medium. As a negative control we added only complete culture medium to the cells. Similar to the Fluc imaging, culture medium was aspirated from the cells, coelenterazine-containing medium was added and Gluc activity was measured immediately for 30 seconds using CCD imaging. Culture medium containing coelenterazine or methanol was left on the cells after imaging. Gluc activity was normalized for the total DNA amount of the well, similar to the Fluc reporter cells as described above.

### Stability of secreted Gluc

Because the highly charged extracellular matrix and avascular nature of the IVD might limit excretion or degradation of secreted Gluc, the stability of Gluc in our *ex vivo* IVD organ was assessed. First, the stability and potential binding of secreted Gluc to the negatively charged proteoglycans of the NP matrix was determined using isolated NP explants that were obtained from goat IVDs. NP explants were isolated by the removal of one IVD endplate and subsequent NP resection. The explants were incubated in either 1 ml PBS or 1 ml PBS containing 0.25 U/ml Chondroitinase ABC (CABC) for 18 hours. CABC is an enzyme that degrades the proteoglycans (an extracellular matrix component of the nucleus), thereby mimicking the early changes in IVD degeneration^37^. After three washing steps of 2 hours in PBS, NP explants were incubated in complete culture medium. Thereafter, we injected NP explants with 50 μl of Gluc-conditioned medium, obtained from 5 × 10^6^ U87-GC cells. The initial Gluc activity of the conditioned medium was 6.5 × 10^6^ RLU, measured in 50 μl medium. Gluc activity in the NP explants was measured by adding 50 μl coelenterazine substrate (1 mg/ml) at various time points for a period of two weeks. Culture medium of the NP explants was refreshed at the days of imaging.

In order to test the possible confinement of secreted Gluc within the complete IVD (including the annulus fibrosus and endplates), we injected IVDs either with 50 μl of Gluc conditioned medium or 0.5 × 10^6^ U87-GC cells. We cultured the discs in the LDCS using the same conditions as described in the section below. Bioluminescence activity was measured at 1, 3, 5 and 7 days post Gluc injection, using the same imaging protocol as described in the methods section “*ex vivo* bioluminescence imaging”. Culture medium of the IVDs was refreshed at the days of imaging.

### Imaging of gADSCs and U87 control cells in the IVD over time

We injected 0.5 × 10^6^ U87-GC, U87-FM cells, gADSC-GC or gADSC-FM in the IVDs (n = 4 for U87, n = 8 for gADSC) and assessed BLI at day 1, 3, 5 and 7 after cell injection, as described in the section “*ex vivo* bioluminescence imaging” above. All discs were cultured in individual culture chambers in the LDCS (Fig. 1b) in DMEM supplemented with, 10% FBS, 1% PFS, 3.5 g/L glucose (Merck, final concentration 4.5 g/L), 25 mMol HEPES buffer (Life Technologies) and 50 μg/ml ascorbate-2-phosphate (Sigma Aldrich). Because IVD cell viability depends on loading, a simulated physiological loading (SPL) condition, consisting of a diurnal dynamical loading (1 Hz sinusoidal) regime as described by Paul *et al.* was applied to each IVD^4^. Culture medium of IVDs injected with gADSCs-GC and U87-GC was collected and secreted Gluc was measured using the luminometer as described in the “*in vitro* bioluminescence imaging” methods section above.

### Histological analysis of IVDs

After the *ex vivo* experiment, we fixed the IVDs in 4% formalin for two weeks and decalcified them in Kristensen’s fluid for another two weeks. Midsagittal slices (3–5 mm) were cut from the midline of the IVD using a scalpel, processed for dehydration and embedded in paraffin. With a microtome, 3 micrometer thin sections were cut starting from the midsagittal line, stained with haematoxylin and eosin (H&E) and analyzed using light microscopy.

### Statistical analysis

Where applicable, data are presented as mean ± standard deviation for each experiment. Different experimental groups were compared and analyzed by a one-way analysis of variance (ANOVA) using SPSS Statistics software version 22 or Graphpad Prism version 6. A p-value < 0.05 was considered statistically significant.

## Results

### Transduction and characterization of gADSCs

**Figure 2:**
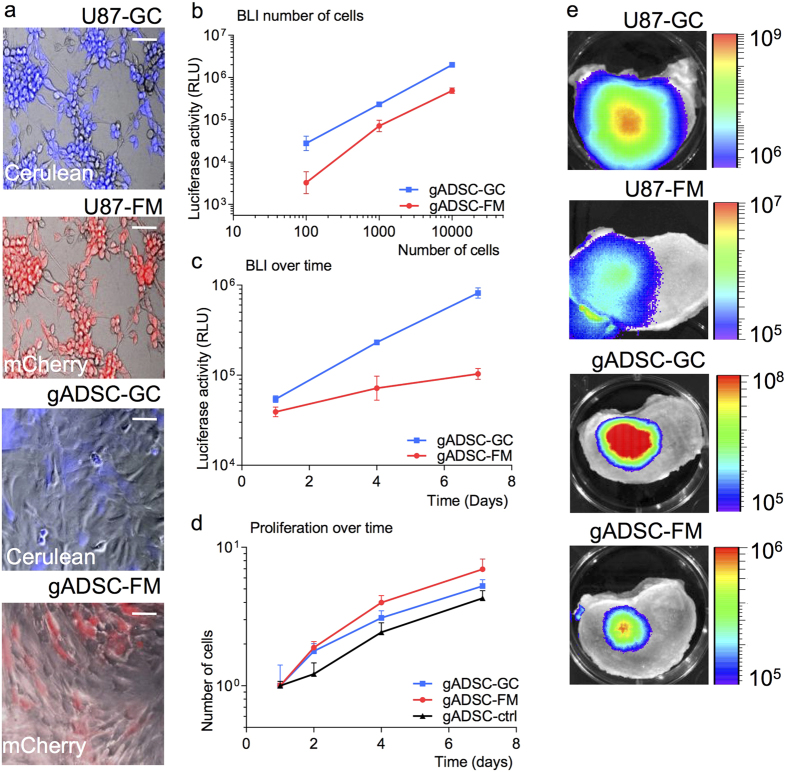
Characterization of goat adipose derived stem cell and U87 reporter cells. (**A**) Fluorescent microscopy overlay images of U87 cells and gADSCs expressing Fluc-mCherry (FM) or Gluc-CFP (GC). Bars represent 100 μm. **(B**–**D**) Fluc reporter and Gluc reporter measurements of gADSC cells *in vitro*. Measurements are in triplicate and error bars represent SD. (**B**) Different amount of cells were plated and one day later, Fluc and Gluc activity was measured. (**C**) Gluc and Fluc activity was measured over the time of seven days. (**D**) gADSC cells were plated and counted over the time of seven days to analyze growth rates. **(E)** Bioluminescence imaging with a CCD camera of an IVD injected with U87-GC, U87-FM, gADSC-GC and gADSC-FM. Imaging was performed directly after injection of the reporter cells. Scale bar represent bioluminescence in relative light units (RLU).

We estimated the lentivirus vector transduction efficiency for both reporters (Fig. 1a) by fluorescence microscopy of CFP or mCherry expression to be ~60% for the gADSCs and ~90% for the U87 cells (Fig. 2a). Expression of the fluorescent proteins in the U87 cells was increased compared to the gADSCs; therefore longer exposure times were needed for a clear visualization of CFP and mCherry in the gADSCs. In different *in vitro* experiments we characterized the expression of Firefly and *Gaussia* luciferase by the transduced gADSCs. Luciferase activity was found to be proportional to the amount of cells (Fig. 2b). In addition, an increase of luciferase activity over time was measured for both gADSC-FM and gADSC-GC (Fig. 2c), indicating cell growth. We confirmed this by counting cells. Fluc half-life is relatively short (about 4 hours^38^) compared to the *in vitro* Gluc half-life (about six days^39^). Since Gluc is also secreted from the cells into the culture medium, it can easily accumulate over time (Fig. 2c). Taken together, this results in a cumulative effect for Gluc and a subsequent steeper slope for Gluc activity compared to Fluc activity.

Microscopic analysis showed no significant effect of the lentivirus transduction on the morphology of the gADSCs. When we expanded the gADSCs for more than four passages, both the transduced and non-transduced cells started to spread out and showed increased stress fiber content. Therefore we decided to use only low passage gADSCs (P < 4), not showing any stress fibers, for all experiments.

Finally, the lentivirus vector transduction did not negatively influence the proliferation rate of the gADSCs over time (Fig. 2d), since the proliferation rate of the gADSC-FM and gADSC-GC showed a similar increase to non-transduced cells (gADSC-ctrl). Comparable results with U87 cells were described earlier^40^. We conclude that lentivirus vector transduction does not alter gADSC morphology or growth properties and we can therefore use both Fluc and Gluc as reporters to monitor gADSC viability and proliferation.

### Imaging U87 and gADSC cells in IVDs

First, we determined if the bioluminescent signal of the cells could be detected by the CCD camera through the IVD endplate. We started by injecting 0.5 × 10^6^ U87-FM or U87-GC reporter cells in IVDs. U87 cells have a high lentivirus vector transduction efficiency and explicit expression of the reporters. We therefore expected these cells to be easily detectable using BLI. Cell injection was directly followed by injection of the respective substrates and bioluminescent signals of both reporter cell lines could be detected in intact IVDs (Fig. 2e). BLI activity for Fluc and Gluc signals differed greatly, with Fluc activity in the order of 10^7^ photons per second (p/s) and Gluc activity in the order of 5 × 10^9 ^p/s.

When we injected 0.5 × 10^6^ gADSCs, we were also able to detect a bioluminescent signal in the IVDs (Fig. 2e). The activity of gADSCs was comparable to the U87 cells signals, with measured activity in the order of 10^7^ p/s for Fluc and 5 × 10^9^ p/s for Gluc. This experiment demonstrated that it is indeed possible to capture the emitted photons externally after passing through the cartilaginous endplate and small part of the vertebral body (~3.5 mm of tissue).

### Cell viability and Fluc and Gluc activity after repetitive substrate addition to reporter expressing cells

**Figure 3:**
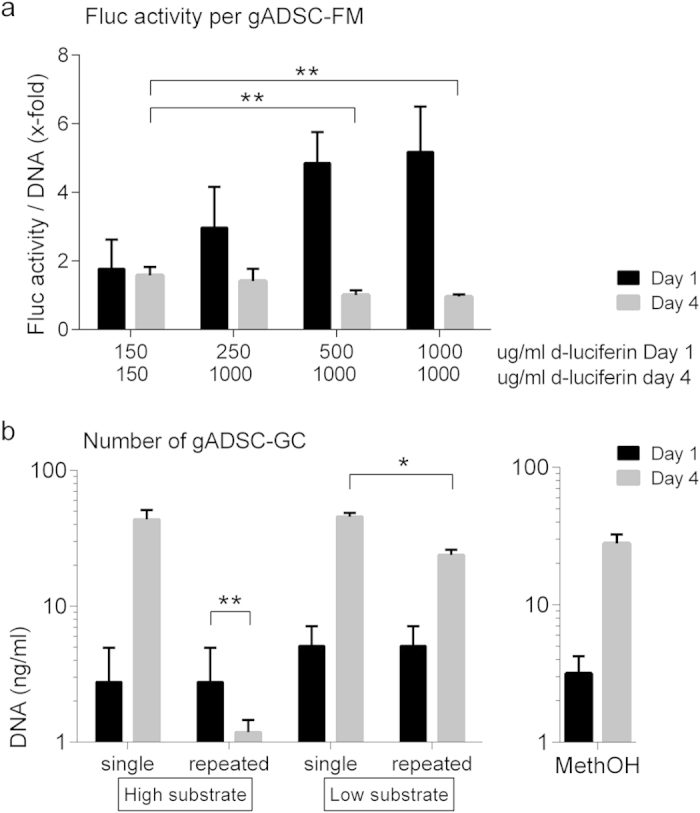
Repeated Fluc and Gluc substrate addition to goat adipose derived stem cell. (**A**) Fluc substrate was repeatedly added to gADSC-FM cells and bioluminescent activity was measured 1 and 4 days after initial plating, with different concentrations of Fluc substrate on day 1 and the same concentration on day 4. Measured activity was normalized for the amount of DNA per well. (**B**) Gluc substrate in two different concentrations (high substrate: coelenterazine 1 mg/ml and low substrate: coelenterazine 5 μg/ml) or methanol was added to gADSC-GC cells. Bioluminescent activity was measured 1 and 4 days after initial plating. DNA contents of all groups were measured. (**A,B**) Experiments were performed in triplicate, data are represented as mean ± SD, *statistically significant different (p  <0.05), **(p < 0.01).

Because the IVD is avascular, excess substrate and reaction products are not readily eliminated from the IVD. We therefore tested the effect of repetitive addition of the luciferase substrates on gADSCs. For the gADSC-FM no significant differences in amount of DNA for all groups were found both on day one and day four (p = 0.166 and p = 0.185 respectively), indicating there is no adverse or toxic effect as a result of repetitive addition of the substrate to the cells (data not shown). However, we did observe a decrease in Fluc activity per cell when the gADSC-FM received the substrate repetitively (Fig. 3a). This decrease in activity was also shown to be dependent on the dose substrate cells received previously. Cells that received 500 or 1000 μg/ml substrate on day one showed a significant decrease in Fluc activity on day four (p = 0.007, p = 0.003, respectively, Fig. 3a). We conclude that, while there is a decrease in Fluc activity after repetitive substrate addition to the Fluc reporter cells, this decrease in activity cannot be explained by substrate toxicity to the cells.

For the Gluc substrate coelenterazine, we observed a significant decrease in DNA content after repeated addition of the high concentration substrate (1 mg/ml) to gADSC-GC (p = 0.001, Fig. 3b). DNA content of the gADSC-GC that repetitively received a low concentration substrate (5 μg/ml) increased over time, although total DNA content was lower compared to the cells that only received a single dose of substrate on day 4. The decrease in DNA content cannot be due to the dissolvent (methanol), since cells receiving an equal amount of methanol but without coelenterazine did not show a decrease in DNA content (Fig. 3b). The total Gluc activity per cell remained constant (data not shown). These results indicate a dose-dependent toxic effect of the Gluc substrate coelenterazine, mainly due to coelenterazine rather than the methanol dissolvent.

### Gluc does not bind to the extracellular matrix of the NP and is not confined within the IVD

**Figure 4:**
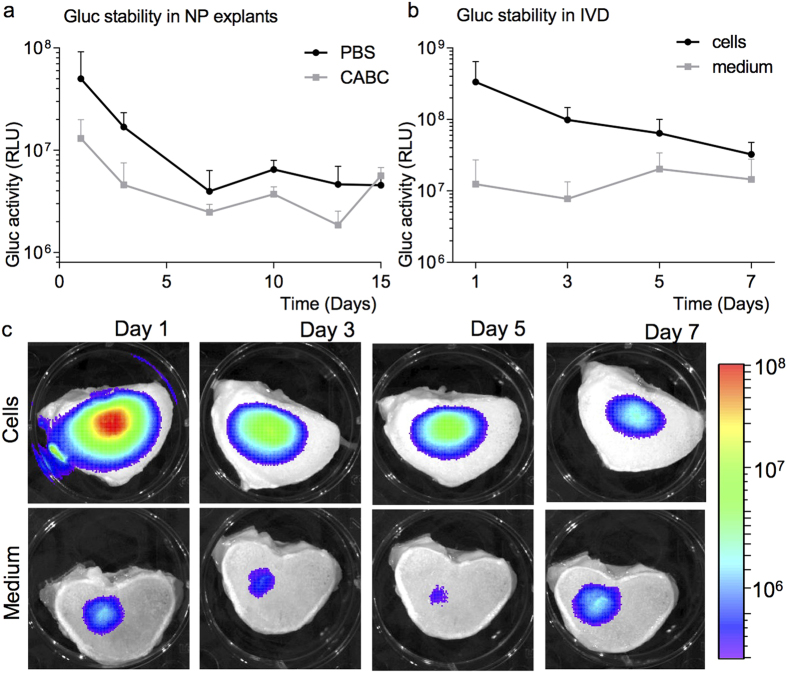
Stability of Gluc in the IVD (**A**) Gluc containing medium was injected and imaged in NP explants with or without pre-treatment with proteoglycan degrading Chondroitinase ABC. (**B**) IVD injection of medium containing secreted Gluc or Gluc expressing U87-GC cells. Gluc activity was determined on specified time points. (**C**) Representative BLI pictures of Gluc activity over time. Scale bars represent bioluminescence (RLU). Experiments were performed in triplicate, data are represented as mean ± SD.

Because secreted Gluc is not removed by circulation in the avascular IVD and is only eliminated by diffusion, knowledge of its stability in our *ex vivo* IVD organ is necessary to correlate Gluc expression to cell viability. To study potential binding of Gluc to the negatively charged proteoglycans in the NP matrix, we injected medium containing secreted Gluc to NP explants and followed Gluc activity for 15 days. When measuring Gluc activity over time, we could not observe a significant difference between the CABC degenerated NPs and the control NPs (Fig. 4a). During the first three days, there was a trend towards higher Gluc activity in the control NPs over the CABC treated NPs, but this non-significant difference was diminished after day three. Therefore we conclude that the possible binding of Gluc to the negatively charged proteoglycans of the nucleus pulposus forms no significant problem in Gluc imaging in IVDs.

To investigate if secreted Gluc is stable and confined within the intact IVDs, we compared IVDs injected with a single dose of medium containing secreted Gluc with IVDs injected with Gluc secreting U87-GC cells. Near background Gluc activity could be observed in the IVDs injected with Gluc conditioned medium for at least seven days (Fig. 4b,c) whereas in the isolated NP explants, activity could be monitored for barely three days (observed on BLI images, data not shown). The Gluc activity of IVDs injected with U87-GC cells clearly remained higher compared to the IVDs injected with medium containing secreted Gluc for the first seven days post-injection (Fig. 4b,c). Gluc expressing cells in IVDs displayed a detectable and quantifiable dynamic range of Gluc activity while the Gluc activity of conditioned medium remained stable but low over time. This result suggests that changes in Gluc activity can be monitored over time and is not significantly hampered by residual Gluc stability.

### Imaging of gADSCs and U87 cells in the IVD over time

**Figure 5:**
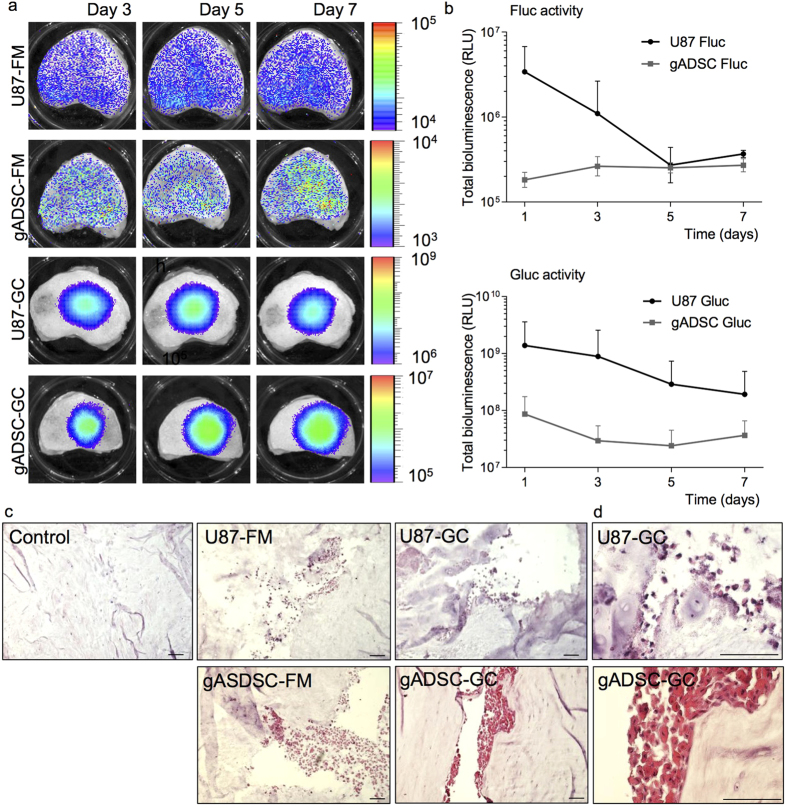
Injection of U87-FM, U87-GC, gADSC-FM and Gluc-GC reporter cells in intervertebral discs *ex vivo*. (**A**) Representative BLI images of luciferase activity of IVDs injected with U87-GC, U87-FM, gADSC-GC or gADSC-FM. Imaging was performed 3, 5 and 7 days after injection of the reporter cells. Scale bars represent bioluminescence (RLU). (**B**) Quantification of bioluminescent signals. Data are represented as mean ± SD, n = 4 for U87 cells and n = 8 for gADSCs. (**C**) Bright field microscopy of H&E staining of IVDs injected with U87-GC, U87-FM, gADSC-GC and gADSC-FM reporter cells. Controls are IVDs without injected cells. Bars represent 100 μm. **(D)** Magnification of selected images from **(C)** showing a more organized pattern and healthy appearance for the gADSCs. Bars represent 100 μm.

For longitudinal monitoring of luciferase expressing cells injected in the IVD, we initially imaged the U87 reporter cells. The U87-GC cells injected in IVDs and cultured in the LDCS showed a strong bioluminescent signal that could be monitored up to the seventh day after cell injection (Fig. 5a). The U87-FM cells injected in the IVD showed a very low (near background) signal that was scattered over the entire disc (Fig. 5a). As with the U87 cells, a strong bioluminescent signal was found for the gADSC-GC whereas the gADSC-FM showed a low background signal (Fig. 5a). The initial signal of the gADSC-GC was lower compared to the U87-GC, however, it was more stable over time (Fig. 5a,b). Gluc could not be detected in the 50 ml culture medium obtained from the individual culture chambers of the LDCS, either for U87-GC cells nor for gADSC-GC injected IVDs. This might be due to the relative large volume of the culture medium compared to the amount of Gluc produced by the luciferase positive cells.

### Clusters of cells can be observed in IVDs injected with gADSC-GC, gADSC-FM, U87-GC and U87-FM cells

We processed the IVDs injected with gADSC-GC, gADSC-FM, U87-GC and U87-FM cells for histological viewing after a culture period of seven days and bioluminescent imaging. We could clearly observe a void within the nucleus pulposus of injected IVDs. No such voids were observed in sections of control discs. Therefore, we consider these voids to be due to injections of cells and substrate in the NP. Inside these voids we could clearly identify clusters of cells that were not found in control IVDs (Fig. 5c,d). Moreover, the gADSCs showed a more organized pattern and intact morphology compared to the scattered distribution for U87 cells, suggesting an increased cell loss within the clusters in the U87 cells (Fig. 5c,d). This corresponds to the reduction of BLI signal for the U87-GC cells.

## Discussion

In this manuscript we aimed to determine the feasibility of using bioluminescent imaging of adipose derived stem cells to monitor and evaluate cell fate and distribution in *ex vivo* culture. In this study the IVD serves as an example of an *ex vivo* organ culture. We evaluated the use of two different luciferases - the intracellular sequestered Firefly luciferase (Fluc) and the naturally secreted *Gaussia* luciferase (Gluc) - for longitudinal cellular imaging within a single sample. We conclude that the Gluc reporter is more suitable for imaging gADSCs in the IVD, as it shows a stable signal for up to seven days. After at least seven days of culture, histological analysis verified the presence of intact cells inside the IVD.

On the cellular level, we observed a lower bioluminescent signal in gADSCs compared to the U87 cell line. This can be expected since tumor cells have a much higher metabolism and activity compared to gADSCs. Also the higher transduction efficiency of U87 cells compared to gADSCs is reflected in the increased BLI signal.

On the reporter level, we found lower BLI signals for the Fluc than for the Gluc reporter in our *in vitro* assays. This could possibly be explained by the differences in gene sensitivity as the *Gaussia* luciferase produces a 2,000-fold higher bioluminescent signal compared to the Fluc reporter^39^. Moreover, the long half-life of Gluc in culture medium results in a cumulative effect^39^ (Fig. 2c).

A striking observation was the finding that luciferase activity of Fluc-expressing gADSCs decreased significantly after repeated administration of substrate *in vitro*. This decrease in activity could not be explained by a toxic effect of the substrate on the cells. We can only speculate about the exact mechanism that causes this decrease, although a change in cell membrane permeability for d-luciferin after initial contact could be a possible cause. Another hypothesis is a negative effect due to a possible accumulation of the residual oxyluciferin after the luciferase oxidation reaction. In contrast to Fluc, a dose-dependent toxic effect was observed for the Gluc substrate coelenterazine *in vitro*.

The *in vitro* experiments, nevertheless, appear to be an aggravated simulation of the *ex vivo* IVD culture. Diffusion in the NP upon injection will lower the local substrate concentration over time, enhanced by mechanical loading of the IVD. The limited effect of Gluc substrate toxicity is supported particularly by the healthy appearance of the gADSCs cells in the IVD (Fig. 5c,d). The decreased viable appearance and morphology of the U87 tumor cells, which have a much higher metabolism and anabolic activity compared to gADSCs, may be the result of a “transition shock” after injection in the hostile environment of low nutrition and oxygenation within the IVD. These harsh conditions may also explain the observed lower BLI signals for the Fluc reporter compared to the Gluc reporter *ex vivo* (Fig. 5a). Both the Fluc and Gluc reporters need O_2_ for substrate conversion, but Fluc is also dependent on ATP and Mg^2+^, elements likely scarce in the avascular IVD due to the minimal exchange of nutrients and other substances^41^,^42^. Furthermore, the intracellular localization of Fluc might also impair photon conversion. For *in vitro* assays, cells are usually lysed before Fluc activity is determined, thus interaction of the enzyme with the substrate may be considered optimal, while in the case of intact cells, either *in vitro* or in the IVD, the substrate-Fluc interaction may be hampered. In contrast, when Gluc is synthesized, it is also secreted into the external milieu, and thus likely enhancing substrate conversion and higher BLI signals.

Possible confounders in our study could be the containment of secreted Gluc in the IVD through binding to the extracellular matrix or entrapment within the intact IVD. We were able to show that Gluc is not efficiently bound in the nucleus pulposus (NP) by the proteoglycans (Fig. 4a). When comparing Gluc expressing cells with Gluc conditioned medium, an increase in BLI signal is observed for the U87-GC cells injected in the IVD (Fig. 4b). Although a direct comparison between Gluc expressing cells and Gluc conditioned medium is difficult, a stable Gluc production by the injected cells is indicated by two results: First, only intracellular Gluc could be present in the cells at the moment of injection, while a clear and high BLI signal could be observed after one day *in situ*, indicating Gluc production by the cells. Secondly, even though the injected Gluc expressing cells represent a relatively larger volume of cells compared to injected amount of Gluc conditioned medium this could not explain the whole 150-fold increase in BLI signal. While the BLI signal of the injected Gluc-conditioned medium remains essentially unchanged and low over time (Fig. 4d), the BLI signal of the Gluc expressing cells decreases. This decrease indicates that dynamic Gluc activity can readily be detected in the IVD and can be the result of a reduction in cell viability. Reduction of BLI signal is predominantly observed in U87 cells, gADSCs showed a far more stable albeit lower Gluc signal (Fig. 5b). This is in line with the decreased cell quality observed in the histological evaluations (Fig. 5c,d). Together, these data indicate that that gADSCs are more able to cope with the hostile IVD environment, and that Gluc can be used as a reporter for stem cell imaging in large animal IVDs.

Although the described method in the current study shows great potential and many alternative applications, some limitations have to be considered concerning the current set-up of this system. The adipose derived stem cells receive the luciferase reporters via transduction by a lentivirus vector. The use of lentivirus vector is an unspecific approach with viral integration in multiple genomic loci and could therefore result in unpredictable genetic modifications and subsequent off-target genetic aberrations and phenotypes of the host cells. However, in multiple lentivirus vector transductions we did not find any signs of differences in cell viability, proliferative capacity or differences in experimental outcomes, implying that this undesired genetic modification is unlikely to occur. Nevertheless, only a complete genomic analysis will provide certainty. Also, the loaded disc culture system is a model system for the intervertebral disc, and although demonstrated to maintain the IVD native properties for over three weeks, it is limited in experimental duration and immune competency compared to the *in vivo* situation. The complexity of the assembly of the loaded disc culture system entails the risk of bacterial contamination after consecutive measurements. Despite these limitations we suggest that the use of an *ex vivo* organ culture allows longitudinal non-invasive real-time monitoring of (stem) cells in organs and complex tissues located in the deeper areas of the body.

Two previous studies reported on the use of bioluminescence imaging for cellular therapies for the intervertebral disc. Omlor *et al.*^43^ used Fluc to monitor cellular activity of autologous mesenchymal stem cells injected in the porcine IVD. Three days after cell injection, Fluc activity was measured by pulverizing the NP and subsequent lysis of the cells. Fluc activity was largely reduced, as shown similarly in the current study. In contrast to our study, lysis of the cells in the study of *Omlor et al.* did not allow further monitoring of cell activity within a single sample. Francisco *et al.*^17^ transduced porcine NP cells with Fluc. These labeled porcine cells were injected in rat IVD explants and Fluc activity was monitored for 14 days. After *in vivo* injection of Fluc expressing porcine cells in the rat IVD, BLI signal was only assessed after 15 hours, without longitudinal follow-up. Not only is a different cell type used (NP cells versus gADSCs in the present study), but the rat IVD used in the study by Francisco *et al.* is also not comparable to the human IVD. The small size of the rat IVD favors nutrient diffusion and BLI signal detection. Furthermore, murine IVDs have different biomechanical properties and also retain their notochordal cells in adult life^44^. These cells are considered stem cell-like NP precursor cells and could therefore create a more suitable microenvironment for the injected reporter cells. Thus, although *ex vivo* BLI imaging in the IVD is not new, the current study is the first to show longitudinal monitoring of gADSCs in large animal IVDs.

The application of *ex vivo* imaging of cells, in particular ADSCs and tumor cells, is of interest for all organ culture models, as it gives insight in the physiology, pathophysiology and therapeutic response. However, the demonstrated cell imaging in intervertebral discs can be interpreted as a worst-case scenario compared to other tissues because of the a-vascularity and subsequent physiological scarcity of nutrients. Therefore, extrapolation of feasibility to highly vascularized organs such as bowel wall, prostate and liver seems highly plausible. However, use of BLI *ex vivo* is influenced by containment of luciferase and toxicity of substrate that should be investigated and considered for every specific tissue.

## Conclusion

In this manuscript we propose live stem cell bioluminescent imaging as a promising tool for the understanding of cellular behavior of large animal organs, studied in 3D organ cultures. We were able to image luciferase-expressing U87 glioma cells and primary gADSCs transplanted in an *ex vivo* organ culture *i.e.* the goat IVD. The Gluc expressing gADSCs show a stable BLI signal over time after injection in the IVD. Furthermore, we investigated a range of possible confounders that could interfere with the bioluminescent imaging of gADSCs in IVDs. Table 1 gives a comprehensive overview of the advantages and disadvantages of Gluc and Fluc bioluminescent reporters. We aim to use this imaging method for longitudinal investigation of the fate and behavior of gADSCs in the IVD, in order to gain a better understanding of their behavior in, and interactions with the native cells and matrix in the IVD and ultimately optimize their regenerative capacities. Also, using this setup one is able to circumvent the technological, ethical and financial challenges that are involved with life animal experimentation and imaging.

## Additional Information

**How to cite this article**: Peeters, M. *et al.* Bioluminescence-mediated longitudinal monitoring of adipose-derived stem cells in a large mammal *ex vivo* organ culture. *Sci. Rep.* **5**, 13960; doi: 10.1038/srep13960 (2015).

## Figures and Tables

**Table 1:**
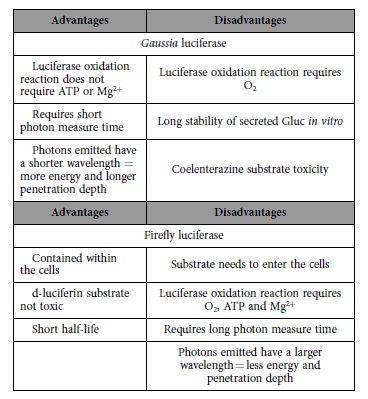
Overview of the advantages and disadvantages of Firefly and *Gaussia* luciferase.
